# Characterization, kinetic, and isotherm data for Cr (VI) removal from aqueous solution by *Populus alba* biochar modified by a cationic surfactant

**DOI:** 10.1016/j.dib.2016.08.051

**Published:** 2016-08-31

**Authors:** Maryam Shahverdi, Esmaeil Kouhgardi, Bahman Ramavandi

**Affiliations:** a Environmental Department, Bushehr Branch, Islamic Azad University, Bushehr, Iran; b Environmental Health Engineering Department, Faculty of Health, Bushehr University of Medical Sciences, Bushehr, Iran

**Keywords:** Adsorption, Cationic surfactant, Cr (VI) removal, Kinetic and isotherm data, *Populus alba* biochar

## Abstract

*Populus alba* is fast and auto- growing tree which profoundly accessible in around the world. The usage of the wastes of this tree would be admirable from environmental and solid waste management point of view. Thus, herein, this data set presents a facile method for providing an adsorbent from wastes of *P. alba* tree. The prepared adsorbent was modified by the cationic surfactant of (C_16_H_33_)N(CH_3_)_3_Br and applied to remove Cr (VI) from aqueous solution. The characterization data of the modified adsorbent were analyzed using FTIR and SEM methods. The information regarding kinetics, isotherms, and thermodynamics of chromium ions adsorption were listed. The data implied that the maximum adsorption capacity of adsorbent to uptake Cr (VI) from aqueous solution was obtained 52.63 mg/g. The acquired data indicated that the adsorption of Cr (VI) by the adsorbent prepared from *P. alba* is an promising technique for treating Cr-bearing wastewaters.

**Specifications Table**

**Table:** 

Subject area	*Environmental Engineering*
More specific subject area	*Adsorption*
Type of data	*Table, image, figure*
How data was acquired	– *The uptake of Cr (VI) by the adsorbent (q*_*e*_*) was determined based on the subtraction of the initial and final concentration of adsorbate.* – *Fourier transform infrared (FTIR) spectroscopy (Shimadzu 4300), scanning electron microscopy (SEM, Hitachi, SU 70) was used for determine the characteristics of the adsorbent.* – *The Cr (VI) concentration measurement was performed by an atomic absorption spectroscopy (AAnalyst 200 Perkin-Elmer).*
Data format	*Analyzed*
Experimental factors	– *The Populus alba biochar (PAB) was prepared from waste of P. alba tree at 350* *°C.* – *The PAB was modified by (C*_*16*_*H*_*33*_*)N(CH*_*3*_*)*_*3*_*Br to produced MPAB* – *Data of MPAB were acquired for Cr (VI) removal from aqueous solution.*
Experimental features	*P. alba biochar for Cr (VI) adsorption from wastewater*
Data source location	*Bushehr University of Medical Sciences, Bushehr, Iran*
Data accessibility	*Data are accessible with the article*

**Value of the data**

• Compare to methods reported in the literature, this data set report a facile and low cost method for Cr (VI) removal from aqueous solution using the *Populus alba* biochar amended by a cationic surfactant.
• The isotherm, kinetic, and thermodynamic data will be informative and useful for predicting and modeling the adsorption capacity and mechanism of chromium removal by the adsorbent from *P. alba*.
• The acquired data will be advantageous for the scientific community wanting to scale up and design an adsorption column with *P. alba* biochar as medium for the removal of Cr (VI)- containing waters or wastewaters.

## 1 Data

**Fig. 1:**
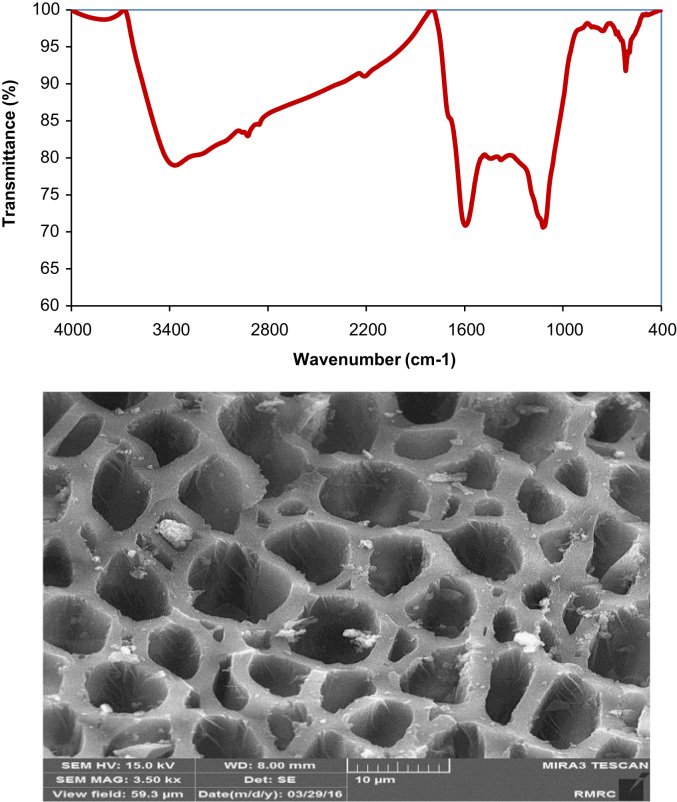
FTIR spectra (upper) and SEM image (downer) of fresh MPAB.

**Fig. 2:**
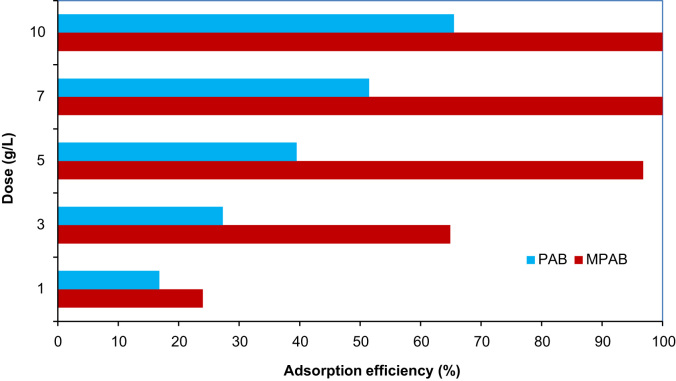
Effect of PAB and MPAB dose on Cr (VI) adsorption.

The FTIR of the fresh MPAB at wave numbers from 400 to 4000 cm^−1^ are given in Fig. 1. The SEM image of prepared adsorbent from *Populus alba* is also illustrated in Fig. 1. The kinetics, isotherms, and thermodynamic parameters were estimated using models listed in Table 1. The data of isotherms and kinetics for adsorption of chromium ions onto MPAB is presented in Table 2, Table 3. Fig. 2 is depicted the comparison data for Cr (VI) adsorption by the MPAB and PAB.

## 2 Experimental design, materials and methods

### 2.1 Materials

All chemical used in this data article such as K_2_Cr_2_O_7_, (C_16_H_33_)N(CH_3_)_3_Br, HNO_3_, and NaOH was purchased from Merck Co. Ltd. The double distilled water was used for preparing working solutions.

### 2.2 Preparation of *Populus alba* biochar

The wastes branches of *Populus alba* tree was gathered from the auto- grown tree beside Symareh River, Iran. Preparation of *P. alba* biochar was done according to method explained in the literature [4], [5]. The sampled wastes branches were first debarked and washed with Symareh River water for removing debris and sand and then shipped to a carpentry workshop. In the carpentry workshop the branches of *P. alba* was cut to achieve 1 cm pieces. After that, by using a Muffle Furnace (350 °C) and a residence time of 6 h, the *P. alba* biochar was provided. The biochar was milled and passed through 25-mesh ASTM-sieve to obtain uniform particles with diameter of 0.707 mm. The uniformed particles i.e., *P. alba* biochar (PAB) were applied in chromium adsorption experiments.

### 2.3 Modification of PAB by [(C_16_H_33_)N(CH_3_)_3_Br] surfactant

The PAB modification was performed following the method described in our previous study [6] with a little change. Briefly, about 20 g of PAB was poured into 180 mL contain 0.06 M (C_16_H_33_)N(CH_3_)_3_Br (surfactant) solution and agitated at 130 rpm for around 10 h. The mixture of PAB- surfactant was then filtered by 0.42- filter papers, and the separated mass was rinsed several times with distilled water to remove unreacted surfactant monomers. The achieved material (adsorbent) was finally fully dried in an oven at 105 °C prior to be used in the Cr (VI) adsorption experiments. The modified PAB was called MPAB.

### 2.4 Design of experiments

#### 2.4.1 General conditions

All tests were done in a batch mode in a 100-mL flask and stirred at 150 rpm in a shaker–incubator instrument (Fan Azma Co., Iran). The initial pH of the solution was regulated to 6 by addition desired amount of 0.1 M HNO_3_. The initial adsorbent dose and solution temperature for all tests was regulated to 7 g/L and 25 °C, respectively. After the sample reached to equilibrium point, the sample was passed through a 0.42 µm- Whatman filter, and the concentration of the residual chromium was determined. The amounts of Cr (VI) adsorbed per gram of MPAB, *q_e_* (mg/g), and the adsorption efficiency of MPAB were obtained as follows [7], [8]:

(1) $$q_{e}=\frac{{\mathrm{Cr}}_{\mathrm{in}}-{\mathrm{Cr}}_{\mathrm{out}}}{M_{\mathrm{ads}}}$$

(2) $$\mathrm{Eff}\left(\%\right)=\frac{{\mathrm{Cr}}_{\mathrm{in}}-{\mathrm{Cr}}_{\mathrm{out}}}{{\mathrm{Cr}}_{\mathrm{in}}}*100$$

where ${\mathrm{Cr}}_{\mathrm{in}}$ and ${\mathrm{Cr}}_{\mathrm{out}}$ (mg/L) are initial and equilibrium concentration of Cr (VI), respectively. *M*_ads_ (g/L) denote the dry mass of PAB or MPAB in the solution.

Duplicate tests were done to ensure the reproducibility of data, and the average measurements are reported herein. Blank tests containing no PAB or MPAB were also prepared.

#### 2.4.2 Isotherms tests

Isotherms analyses were done with Cr (VI) concentrations of 5, 10, 30, 50, 70, and 100 mg/L and contact time of 8 h. The isotherm models of Freundlich, Langmuir, and Temkin was used for isotherm evaluation (see Table 1).

#### 2.4.3 Kinetic tests

Kinetic tests were done using a given initial concentration (50 mg/L) for contact times of 0, 5, 10, 20, 40, 60, and 80 min. The kinetic models of pseudo first- and pseudo second- order were used for kinetic evaluation.

#### 2.4.4 Thermodynamics tests

The thermodynamics of Cr (VI) adsorption by MPAB was performed at solution temperature of 25 °C (298 K) and thermodynamics parameters was acquired using an estimated change in *ΔG*°, *ΔH*°, and *ΔS*° as defined in Table 1.

#### 2.4.5 Effect of MPAB and PAB dose on Cr (VI) removal

The adsorption efficiency of MPAB and PAB was compared at conditions of solution temperature of 25 °C, initial concentration of 50 mg/L, contact time of 60 min, and pH of 6.

### 2.5 Analytical methods

The fresh MPAB samples were pressed into tablets with KBr powder and then were detected by Fourier transform infrared spectrometer apparatus (Shimadzu 4300, Japan) with the scanning range from 400 to 4000 cm^−1^. The morphological characterization of the fresh MPAB was done by scanning electron microscopy (SEM, Hitachi, SU 70). The measurement of residual Cr (VI) concentration in solutions was carried out by using an atomic absorption spectroscopy (AAnalyst 200 Perkin-Elmer). The solutions pH analyses were performed using a pH meter (METLER TOLEDO FE20).

The correlation coefficients (*R*^2^) and standard deviation (*SD*) of the two measurements was applied to assess the suitability of the kinetic and isotherm models. SD was obtained as follow:

(3) $$\mathrm{SD}=\sqrt{\frac{1}{n}\sum_{i=1}^{n}{\left(M_{i}-\overset{¯}{M}\right)}^{2}}$$

where *M*_1_, *M*_2_, …, *M_n_* are the acquired values of measurements, $\overset{¯}{M}$ is the mean value of measurements, and *n* is the sample size.

## Figures and Tables

**Table 1:** Isotherm and kinetic and thermodynamic models/equations used in this data article [1], [2], [3].

Model	Functional form	Plotting
Langmuir	$\frac{q_{e}}{q_{\max}}=\frac{K_{L}C_{e}}{1+K_{L}C_{e}}$	$\frac{1}{q_{e}}\mathrm{vs}\frac{1}{C_{e}}$
Freundlich	$q_{e}=K_{f}C_{e}^{1/n}$	ln $q_{e}\;\mathrm{vs}\;\log\;C_{e}$
Temkin	$\frac{q_{e}}{q_{m}}=\frac{R.T}{b}\ln\left(C_{e}k_{T}\right)$	$q_{e}\;\mathrm{vs}\;\ln\;C_{e}$
Pseudo first order	$\frac{\mathrm{dq}}{\mathrm{dt}}=k_{1}(q_{e}-q_{t})$	ln ${(q}_{e}-q_{t})\mathrm{vs}\;t$
Pseudo second order	$q_{t}=\frac{q_{e}^{2}k_{2}t}{1+q_{e}k_{2}t}$	$\frac{t}{q_{t}}\mathrm{vs}\;t$
Thermodynamic equations	*ΔG*°= −*RT* ln *K_Th_*; *ΔG*°= *ΔH*°− *TΔS*°; ln *K_T_*= (*ΔS*°/*R*)−(*ΔH*°/*RT*)	ln *K_t_ vs* 1/*T*

*q*_*max*_ = maximum adsorption capacity, *k*_*L*_= Langmuir constant, *k*_*f*_ and *n* = Freundlich constants; and *k*_*T*_ and *b* = Temkin constants, *k*_1_ = rate constant of pseudo first order model, *k*_2_ = rate constant of pseudo second order model, *q*_*t*_= adsorbed amount at any time, *q*_*e*_ = adsorbed amount at equilibrium, *R* = universal gas constant, *T* = absolute temperature (K), *ΔG°*= Gibbs free energy change (kJ/mol), *ΔH°*= enthalpy change (kJ/mol), *ΔS°*= entropy change (kJ/mol K), and *K*_*T*_= thermodynamic constant (mL/g).

**Table 2:** Kinetics data for Cr (VI) adsorbed onto MPAB.

Parameter	Value

*q*_*e*, exp_ (mg/g)	2.212

Pseudo first order	
*q*_*e*,cal_ (mg/g)	1.362
*k*_1_ (min^-1^)	0.020
*R*^2^	0.984
*SD*	0.089

Pseudo second order	
*q*_*e*,cal_ (mg/g)	2.101
*k*_2_ (g/mg.min)	0.029
*R*^2^	0.946
*SD*	0.028

**Table 3:** Isotherms and thermodynamic data for Cr (VI) adsorbed onto MPAB.

Parameter	Value
*q*_*e*,exp_ (mg/g)	49.111

Freundlich	
*K_f_* (L/g)	22.83
*n*	4.05
*R*^2^	0.875
*SD*	0.024

Langmuir	
*K_L_* (L/mg)	0.157
*q*_max_ (mg/g)	52.63
*R*^2^	0.994
*SD*	2.330

Temkin	
*K_T_* (J/mol)	5.29
*b* (J/mol)	297.87
*R*^2^	0.965
*SD*	5.746

Thermodynamic parameters (at 298 K)	
*∆G*° (KJ/mol)	−2.45
*ΔS*° (KJ/mol)	0.0019
*∆H*° (KJ/mol)	−1.852
*R*^2^	0.986
